# Assessment of Iterative Closest Point Registration Accuracy for Different Phantom Surfaces Captured by an Optical 3D Sensor in Radiotherapy

**DOI:** 10.1155/2017/2938504

**Published:** 2017-01-09

**Authors:** Gerald Krell, Nazila Saeid Nezhad, Mathias Walke, Ayoub Al-Hamadi, Günther Gademann

**Affiliations:** ^1^Institute for Information Technology and Communication Engineering, Otto-von-Guericke University Magdeburg, Universitätsplatz 2, 39016 Magdeburg, Germany; ^2^Clinic for Radiotherapy, Otto-von-Guericke University Magdeburg, Leipziger Straße 44, 39120 Magdeburg, Germany

## Abstract

An optical 3D sensor provides an additional tool for verification of correct patient settlement on a Tomotherapy treatment machine. The patient's position in the actual treatment is compared with the intended position defined in treatment planning. A commercially available optical 3D sensor measures parts of the body surface and estimates the deviation from the desired position without markers. The registration precision of the in-built algorithm and of selected ICP (iterative closest point) algorithms is investigated on surface data of specially designed phantoms captured by the optical 3D sensor for predefined shifts of the treatment table. A rigid body transform is compared with the actual displacement to check registration reliability for predefined limits. The curvature type of investigated phantom bodies has a strong influence on registration result which is more critical for surfaces of low curvature. We investigated the registration accuracy of the optical 3D sensor for the chosen phantoms and compared the results with selected unconstrained ICP algorithms. Safe registration within the clinical limits is only possible for uniquely shaped surface regions, but error metrics based on surface normals improve translational registration. Large registration errors clearly hint at setup deviations, whereas small values do not guarantee correct positioning.

## 1. Introduction

Tomotherapy combines a CT scanner with a computer-controlled radiation beam collimation system at the treatment machine [1] to precisely target tumors sparing healthy tissue. The system installed in Magdeburg hospital is a Tomotherapy HD system which enables helical and fixed radiation in one single system. A helical slit delivers radiation with most conformal image guided radiotherapy (imrt). The x-ray source rotates in a helical path around the patient in order to acquire a 3D image. The same x-ray source is used as treatment beam. This source is rotating in a helical pattern around the patient, while the intensity of beam is modulated according to the tumor shape using “tungsten leaves.” These leaves create thousands of beam elements, called “beamlets” [2]. The radiation is delivered by a discrete-angle, nonrotational method sequentially moving the treatment table from the center of the system and for each angle of the gantry. Optical sensors provide an additional tool to verify the precise positioning of the radiation target relative to the treatment machine. The actual position in the treatment fraction is compared with the desired position given by a previously recorded reference surface. The reliability of such ICP-based algorithms is investigated in this paper by comparing the results of the implementation by the optical sensor with selected popular algorithms.

## 2. Methods and Materials

### 2.1. Optical Sensors in Tomotherapy

Nowadays, image guided methods are increasingly used in radiotherapy [3–11]. The target regions of irradiation and the intended dose distributions are mostly defined on the basis of CT scans. Then an irradiation plan is created which involves the placement of the patient with regard to the treatment machine and the control of the irradiation beam. The main aim is to hit the tumor with sufficient energy and to protect healthy tissue and organs as much as possible against irradiation at the same time. Exact placement of the patient in the irradiation session is therefore very important. In addition to correct positioning by integrated CT in the treatment machine, optical sensors can capture surface data.

Optical surface sensors hence provide an additional tool for contact-less verification of patient position and are now getting into clinical practice after a long-time development and use for scientific purposes.

**Figure 1:**
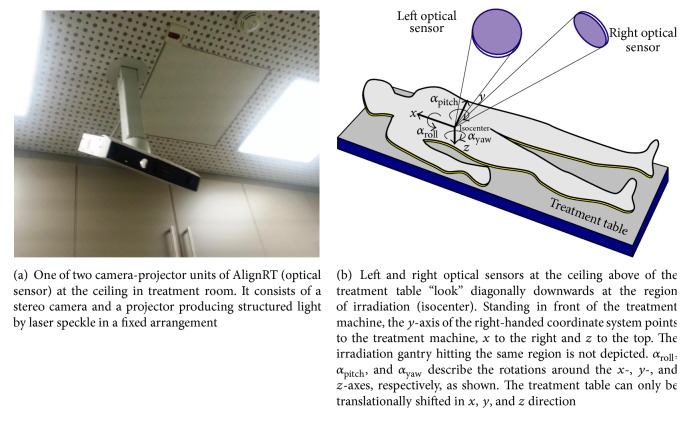
Optical sensor in radiotherapy.

Our Tomotherapy HD accelerator unit (Accuray, USA) is combined with an AlignRT (VisionRT Ltd., London, UK) system and consists of two pods laterally positioned corresponding to the virtual isocenter in front of the Tomotherapy bore. The virtual isocenter lies 700 mm outside in front of to the real radiation isocenter of the machine. The distances of the two pods in respect to the virtual isocenter are about 2.0 m. The two pods are tightly mounted at upper ceiling of the treatment room. They are right and left of the Tomotherapy couch. Each of the two units each consists of two cameras (stereo) and a speckle projector producing structured light (Figure 1(a)) to generate a 3D model of the patient's surface by close-range photogrammetry (triangulation) [12]. The unit also includes a texture camera for visualization purposes, which, however, is not used for alignment [3]. The AlignRT parameters of the optical system are estimated and verified by daily calibration using a calibration plate that is aligned with a virtual laser isocenter in front of the real isocenter. Real-time capability of the AlignRT system relates to the ability of the sensor to capture surface data fast enough to even follow typical human motion caused by respiration, for example. Although tracking of the surface is fast enough to meet these requirements, the first registration takes longer and is therefore usually done offline.

The units are installed at the ceiling in the treatment room above of the treatment table and in front of the irradiation gantry in such a way that they capture the body surface at the target region (isocenter) diagonally downwards from two directions in order to reduce occluded regions (Figure 1(b)). The radiation gantry of the treatment machine is situated on one point of a circle around the isocenter parallel to the *xz*-axes. The *x*, *y*, and *z* position of the treatment table at the radiation gantry can be shifted computer-controlled with an accuracy of about 0.5 mm. Rotation of the treatment table is not possible, although in real situations rotational displacement of the patient must be expected.

An optical sensor of the considered type estimates a distance map related to a measured surface by finding correspondences in images taken from two or more directions by photogrammetric methods [12, 13]. A typical scheme is first to calculate a standard view of the recorded images by rectifying them on the basis of the camera parameters obtained by the previous calibration. Finding the correspondences in the images gives the disparity maps which describe the parallax caused by the distance between the cameras of one optical sensor. Together with camera calibration parameters the depth map is then calculated from the disparity map which can be considered as a mesh of 3D points or as a point cloud. Because the depth values are calculated corresponding to the pixel grid neighborhood relations are directly given and a mesh grid, for instance, consisting of triangles, can be easily calculated. The surfaces of the two optical sensors of AlignRT are merged in one data file. At the transition of the surface data of one sensor to the other some overlapping or gaps may occur. The software of the optical sensor handles these problems and produces a single more or less closed surface of triangles out of the data of the two sensors. Details are not given by the manufacturer. Rigid registration parameters for different snapshots of captured 3D surface data can be calculated by the propriety software.

Optical sensors provide an additional modality to estimate the patient position on the basis of the outer body shape without increasing radiation load. Here we consider the application of the optical sensor without use of additional markers. The surface data captured is therefore a point cloud or a mesh grid corresponding to pixels of the image sensor. In such an unconstrained setting without markers, we just know that a surface point estimated by the optical sensor belongs to some corresponding point of the surface in the voxel image captured for definition of the 3D planning volume. But the exact position of this corresponding point on the surface in this image, which is usually a CT scan, is not directly given. This correspondence can only be estimated out of the form of the reference surface if it is successfully matched with the surface to be tested. In this way corresponding regions are registered and transformation matrices are calculated representing a measure for the deviation between a reference and a test surface.

Two operation modes of the surface sensor are distinguished in clinical practice: the static setup verification of patient (single-frame surface acquisition) and the tracking of patient motion (continuous dynamic surface acquisition mode), for example, caused by respiration [3]. In the latter case the solid assumption is appropriate if the time step from one surface capture to the next is small enough.

For real patients, the shape may also have been changed in the static setup phase introducing additional uncertainties in ICP registration. Respiratory motion of a patient surface blurs the registration result which is an additional effect and should not be mixed with the uncertainty of ICP registration. Motion blur in the 3D measurement results may cause additional problems. Our paper therefore considers the static case and shows that even with solid phantoms uncertainties remain depending on the individual shape.

### 2.2. Principles of ICP Algorithms

Surface registration assumes that two or more surfaces can be matched by a geometrical transformation. Resulting transformation parameters then describe the deviation between the surfaces for a correct registration. In case of radiotherapy, the optical sensor should ensure that the patient is placed according to the irradiation plan. The desired position is usually defined on basis of the CT data set. If we want to compare a test surface measured by optical sensors with the target position we would have to extract the corresponding surface data out of the CT as a reference. Tomotherapy gives us another option: because a CT is directly available at the treatment machine together with the optical sensor, we are able to bring the patient exactly to the desired position by the CT modality. The surface scan of the optical sensor at this position can be considered as a reference (target position). In later treatment sessions, the memorized reference position can then be used to bring the patient back to the desired position by the measured test surface. Also during the irradiation itself the correct position can be verified by the optical surface scan because, in contrast to CT, optical data are available during the whole treatment.

#### 2.2.1. Known Point-to-Point Correspondences

The alignment of surfaces is much more simple and unique in case of known correspondences between reference and test surface. This assumes that registered *n* points of the reference surface *p*_*i*_ = *p*(*x*_*i*_, *y*_*i*_, *z*_*i*_) with index *i* = 1,…, *n* and spatial coordinates *x*_*i*_, *y*_*i*_, *z*_*i*_ are ordered in pairs with *n* points *q*_*i*_ = *q*(*x*_*i*_, *y*_*i*_, *z*_*i*_) of the test surface.

A linear transformation matrix *R* and a translation offset vector *t* aligning reference and test surface are directly estimated by minimization of the sum of the squared error:

(1) $$\begin{array}{c} E\left(\;R,t\;\right)=\overset{n}{\underset{i=1}{\sum }}{\left\|\;p_{i}-Rq_{i}-t\;\right\|}^{2}⟶Min, \end{array}$$

which means that the (geometric) distance between the reference surface and the transformed test surface should be as small as possible. When the correct correspondences are known a unique solution for *R* and *t* for given *n* = *N* point pairs or a solution in the least square sense for *n* > *N* directly yields. When limiting to an affine transform, the linear transform matrix with *N* ^2^ parameters modifies to a rotation matrix with *N* rotation angles resulting in a 2*N*-dimensional optimization problem (together with the *N* translation parameters). Such a nonlinear equation can generally only be solved iteratively or with a linearized approximation assuming small angles.

#### 2.2.2. Unknown Point-to-Point Correspondences

In general, without fiducial markers, no direct correspondence between points of the surfaces is given and also the number of points to be registered may be different. In this situation, the transformation matrix cannot be determined immediately. In ICP algorithms, the closest point in the reference is considered as the corresponding point of the test surface iteratively adapting the transformation matrix in each iterative step. Sophisticated search strategies exist in order to avoid a complete search between the two surfaces. The transformation in each iterative step does not align the two surfaces perfectly but brings them closer to each other in the converging case.

Registration fails in the case of growing deviation between the two surfaces in the iterative steps. When converging, the registration is terminated by a certain criterion such as size or gradient of the deviation error; that is, a certain registration error generally remains for real measurement data. ICP algorithms perform a local search on the error surface describing the deviation of the actual measurement from the target and estimate translation and rotation matrices as registration parameter. They converge well when a unique error minimum exists, but problems may arise when trapping in side minima occurs. In the latter case, registration is inaccurate or fails completely.

Reference [14] gives a good overview on ICP algorithms for technical applications with three synthetically generated scenes providing test surfaces to evaluate the variants. In this way the correct transform is known exactly.

ICP algorithms can be divided into different phases. According to [14], typical ICP algorithms perform the following steps.

*(1) Selection of the Source Points (Measurement).* Different criteria for handling point clouds are considered. Using the complete set of points to find the transformation parameters might be slow; therefore the data could be randomly or regularly subsampled. Another strategy is to extract significant points at edges or corners where the information is concentrated. This method of sampling requires preprocessing but it reduces the number of required points improving accuracy and efficiency of the algorithm.

*(2) Matching.* This step is the most costly step in ICP algorithm. There are different methods such as building a kd-tree search to speed up finding corresponding closest points. The simplest idea is finding the closest point in the other point cloud for each point. The result of this method is generally stable but it computes slowly. Another method to find the correspondence is “shooting” along the normal of each point to the other point cloud. The intersection of the normal and point cloud is considered as the corresponding point [14]. There is a faster method to match the correspondence which is projection based matching. In this method the points lying on the line of sight of one of the cameras are considered correspondent. In this case the result is good if two cameras are close enough [14].

*(3) Weighting.* The matched point pairs can be weighted with regard to certain additional criteria describing the similarity of the corresponding region such as color, distance, curvature, or direction of tangent normal [14]. To this end, the error metric is multiplied by a weighting factor depending on the specific criterion.

*(4) Rejection.* Rejection of certain point pairs can be implemented after each matching step in order to improve alignment. This can be done in the phase of search for the closest neighbor. Several rejection methods have been proposed in different studies [14]: rejection of those point pairs with a distance greater than a user specified limit, rejection of a certain portion of point pairs with largest distance, and rejection of point pairs inconsistent with neighbor pairs (rigid transform).

*(5) Error Metric and Minimization.* This step is the last step of ICP algorithm which measures the error between the point clouds and tries to minimize the distance between two point clouds. Mostly either a “point-to-point” or a “point-to-plane” error metric is applied. In the first case, if *p*_*i*_ is a source point and *q*_*i*_ the corresponding point in the target point cloud and *M* is the transformation matrix, then the sum of squared distances has to be calculated and minimized [2]:

(2) $$\begin{array}{c} M_{min}=\underset{M}{\arg\min}\overset{i}{\sum }{\left(\;M\cdot p_{i}-q_{i}\;\right)}^{2}. \end{array}$$

Closed form solutions for this kind of error metric exist, such as singular value decomposition (SVD), dual quaternions, quaternions, and orthonormal matrices. Accuracy and stability of these methods have been evaluated by [15].

In general, point-to-plane error metric converges better than the point-to-point error metric [16]. It minimizes the sum of squared distances between source points and the tangent plane at the target point which is orthogonal to the unit normal vector of that point. Mathematically, if *p*_*i*_ is a source point and *q*_*i*_ is the corresponding point in the target point cloud and *n*_*i*_ = *n*(*x*_*i*_, *y*_*i*_, *z*_*i*_) is the normal vector at *q*_*i*_ then the ICP algorithm estimates the rigid transformation matrix by the minimizing function

(3) $$\begin{array}{c} M_{min}^{norm}=\underset{M}{\arg\min}\sum {\left(\;\left(\;M\cdot p_{i}-q_{i}\;\right)\cdot n\;\right)}^{2}. \end{array}$$

Because no closed form solutions for point-to-plane error metric exist it is usually solved iteratively by nonlinear methods such as Levenberg-Marquardt or it can be linearized considering some approximation for rotation matrix *R*, such as replacing sin⁡*θ* by *θ* and cos⁡*θ* by 1. The problem of the point-to-plane error metric is that it is sensitive to noise and that it does not converge well if the distance between two point clouds is large [15, 17].

The ICP algorithm can vary by changing the methods in each step to improve the performance with regard to speed and stability depending on the amount of noise and outliers the algorithm can deal with.

### 2.3. Selected ICP Algorithms for Registration

Four different, under BSD license available, ICP implementations in Matlab have been compared with the proprietary software of AlignRT for surface registration of phantoms. We have chosen the same software platform because one criterion was the option to compare the speeds. We assumed that the implementations belong to the most popular ones. They all meet the same general ideas of ICP registration and present the variety of unconstrained methods (without markers or using colors). We found that the four chosen ICP algorithms are well suited to be compared with the method applied by AlignRT. An interesting extension of work would be to include new approaches to point registration such as described in [18].

*(1) Wilm's Algorithm [2, 19].* Point clouds are aligned by considering the complete points set. The program finds the nearest neighbor by a kd-tree search which considerably increases the speed of matching. Point-to-point or point-to-plane error metric can be selected by parameter setting. AlignRT uses a similar point-to-plane metric as follows from the communication with the manufacturer.

*(2) Kroon's [20], Modified.* This program uses a finite difference model to align the point clouds. The finite difference method also supports the transform types of resizing and shearing. Several optimization functions are included for minimum search. We added a global search approach by generating different start points using a scatter-search method to improve the results. All starts points are evaluated and the points which are unlikely to improve the minimum are rejected.

*(3) Renoald's [21].* It is a simple ICP implementation which uses all the data points. It first finds the corresponding points by creating a Delaunay tessellation of points in a model to search for the closest point. Then it calculates the initial transformation matrix by singular value decomposition (SVD) and applies this to the target point cloud. The transformation matrix is updated iteratively until no more correspondences can be found.

*(4) Bergström's [22, 23].* It is similar to the Renoald's algorithm with the main difference that, after matching corresponding points, the point pairs are weighted by the maximum point distance. Levenberg-Marquardt algorithm is directly applied to minimize the squared sum of the distances of closest points.

Most of the implementations allow choosing among modes and modifying parameters. The best configuration for this experimental setup has been investigated and shown in Table 3. The above given references give further details.

### 2.4. Related Works

Reference [4] compared suggested setup correction with a second and independently operated marker-based optical system with an anthropomorphic plastic phantom and healthy volunteers. They found alignment accuracies of about 1 mm for translation and 0.5° for rotation as an average. Using markers is more invasive and time consuming but in general safer than unconstrained registration.

Extensive research has been done on the development of surface sensors. The general ideas are shown in works as [12, 13]. Reference [24] deals with the simulation of photogrammetric triangulation in order to develop the algorithms without need of acquisition of additional camera data.

Reference [3] investigated the temporal stability of alignment accuracy in the context of respiratory motion in an operation mode where the sensor is triggered by the breathing phase. A rigid, flesh-colored mannequin torso phantom has been used. In this approach, the optical sensor is combined with an infrared-based marker system for gating the breathing state and a motorized mechanical stage. Measured surface data has been compared with surface extracted from CT as a reference. High stability and errors in the submillimeter range and less than 1° have been reported. Additionally, the accuracy of recommended patient realignment has been evaluated for 54 random shifts of the treatment table. In our investigations we focused our attention on the influence of different types of phantoms in order to learn how curvature influences the registration reliability.

Reference [14] gives helpful results how existing ICP algorithms converge for synthesized surfaces. Also different sampling strategies for selection of registration points have been considered. But for clinical practice it is important to verify these theoretical results with the real situation for data of an existing optical sensor.

Reference [7] evaluates a 3-Dimensional Surface Imaging System for Guidance in DIBH Therapy. Setup data based on captured 3D surfaces by the same surface imaging system as we used was compared with setup data based on cone beam computed tomography (CBCT) and evaluated with regression based methods. It was found that in the context of breast cancer treatment 95% of the deviations less than 0.4 cm detected by the optical sensor were less than 0.66 cm in the other mode of CBCT. A comparison of megavoltage CBCT based registration and of AlignRT based registration to its own particular reference is subjected to certain time constraints. A CT scan itself as a possible reference and the local megavoltage CBCT scan on the Tomotherapy unit is usually a time-consuming procedure.

Reference [6] reports on two commercial optical sensors (surface imaging systems) and compares them with the actual adjustments in patient positions made on the basis of megavoltage CT scans. The deviations between the proposed correction of the optical sensor and the subjectively best alignment of an expert have been statistically evaluated. Tests have been performed on an Alderson phantom and on patients at head/neck, pelvic, and chest regions. It was found that the optical sensors can support patient positioning mainly at pelvic and chest regions because immobilization of the patient by special masks is not possible as in the case of head and neck region.

Generally, the AlignRT system is usable on nearly all possible patient regions. Some papers deal with clinical applications of optical sensors to different patient regions. Besides classical patient body region dependent applications, the frame- and mask-less cranial stereotactic radiosurgery is a new application field. The comparison of breath induced surface movements with different registration modalities is subjected to different time constants of the acquisition devices. The verification of DIBH (depth inspiration breath-hold) techniques with optical systems, as the AlignRT system, is a new emerging procedure in the clinical practice.

A feasibility study for the usability of the AlignRT system to frame- and mask-less cranial stereotactic was presented by [8]. The presented technique shows the potential of head mold and surface monitoring to use in stereotactic treatments. The accuracy of the surface imaging motion tracking system during the stereotactic treatment was verified. The results were additionally tested on the standard optical guidance platform technique (kVCT by Varian).

Work [9] describes a clinical analysis of fifty patients with the AlignRT system in comparison to megavoltage portal imaging. Daily alignment with the 3D optical imaging system was found to be valuable for reducing setup errors in comparison to skin markers. Particularly the anterior-posterior alignment directions were with the optical system noticeably better.

The possible synchronization of a classical CBCT system with the AlignRT has been shown by [10]. An image guided method for the synchronization of the X-ray projections is synchronized with optically sensed surface during using CBCT without any further hardware requirements. The proposed method can by generically applied to any configuration of the CBCT and optical imaging systems and also be used for extracranial tumor tracking.

## 3. Generation of Test Surface Data

In order to generate surface data we focused our work on rigid phantoms because we are mostly interested in pure accuracy of the sensor together with the ICP algorithms in the ideal case. The investigated ICP algorithms do not treat shape variations which is a motivation for using solid phantoms instead of real cases. The influence of motion of real human bodies, caused by respiration for instance, is considered by other papers (e.g., [10, 11]).

### 3.1. Test Phantoms

Because the contour characteristics of a surface is important for a safe registration, specially designed phantoms of different surface types have been investigated. To this end, dedicated phantoms have been designed or selected with a size approximately covering the measurement volume of the optical sensor of about 0.1 m^3^. In this study, four different phantoms have been measured by the optical sensor in order to generate point clouds for the evaluation of the ICP algorithms.

*(i) Plane.* It is a simple plane horizontally placed on the treatment table. The main idea is to check the accuracy of the optical sensor with regard to vertical shift of the treatment table (*z* direction).

*(ii) 3plane.* It consists of two planes and an edge especially built to allow a unique matching with respect to all *x*-*y*-*z* space coordinates.

*(iii) Bowl.* The bowl phantom is more curved than a plane, but ambiguities with regard to rotations must be expected.

*(iv) Torso.* By the torso of a mannequin, a shape typical for the human body has been simulated. The curvature of the torso phantom is more ambiguous in the cranial-caudal (*y*) than in the dorsal-ventral transverse motion direction.

**Figure 2:**
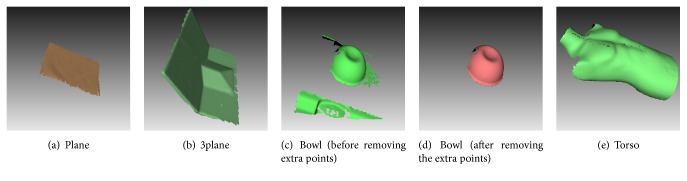
Surfaces of selected phantoms captured by the optical sensor AlginRT showing typical problems of real measurement data: (a) measurement noise and systematic errors; (c) extra points not belonging to the object of interest; (b) and (e) seam from fusing the two surfaces of left and right optical sensors.

The phantoms have been coated by white painting or textile to produce a surface that can be well captured by the cameras of the optical sensor when illuminated by the speckle projector. Measured point clouds of these phantoms are shown in Figure 2. As visible in Figure 2(c), the measured surfaces contained some points of the background (e.g., of the treatment table). Such extra points obviously not originating from the phantoms have been manually removed for the data of all phantoms. As an example, Figures 2(c) and 2(d) show the bowl surface before and after removing the extra points, respectively.

### 3.2. Test Setup

The above described ICP algorithms have been tested with surface data of the selected phantoms (Figure 2) moved to well-defined positions. First, the optical sensor AlignRT has been calibrated according to the instructions of the manufacturer. Then the phantoms have been placed on the treatment table and a surface scan at the origin has been captured. This surface scan at central (zero) position of the treatment table served as a reference to compare with surface scans at other positions. To this end, the phantoms have been translationally shifted by the treatment table in the directions *d* = {*x*, *y*, *z*} by distances of *s*_*d*_ = {0.5,1.0, ±10.0,20.0} (mm). For the plane, translation was only done in *z* direction (*d* = *z*) because tests confirm the obvious fact that a motion in *x* or *y* direction cannot be detected if the plane phantom is placed in parallel to the *x*-*y*-axes as we did.

**Figure 3:**
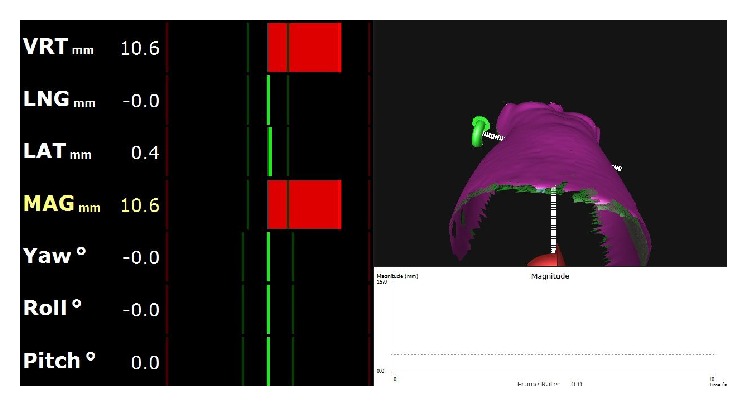
An example of the AlignRT monitoring screen seen during measurement of the torso phantom and vertical shift of the treatment table *s*_*d*_(*z*) = 10 mm. The reference surface is shown in pink and the measured surface in green. The suggested linear translations (vertical, lateral, and longitudinal) and rotations (yaw, pitch, and roll) are shown by numbers and colored bars on the left together with the RMS value (called magnitude MAG). The white graph is used to display a time series of the RMS values (not used in our experiment).

Figure 3 gives an example on how the operator sees the situation on the monitoring screen of AlignRT. It shows the estimated misalignment for translation and rotation in mm and °, respectively, by numbers with one-digit accuracy after the comma and by bars. At setup, the therapists attempt to minimize the shifts (by minimizing the length of the bars) [11]. The surface data is exported as object files and used for the registration by the other ICP algorithms.

After an initial phase, real-time surface tracking is possible with the AlignRT system. AlignRT system delivers sufficiently fast displacement estimation for most medical indications of about 10 frames per second. Acceptable speed relates mainly to the time needed for an initial alignment which should not exceed about a second in order to be acceptable in clinical routine.

Therefore two requirements result with regard to the speed: the alignment time should not be much longer than a second because more cannot be accepted in clinical routine.

In case of dynamic tracking, the speed demands arise by the typical patient motion to avoid subsampling on the one hand and to ensure that shape variations between two time steps can be neglected for rigid registration. In the ideal case, the registration should be faster than the surface sensor in order to avoid reduction of frame rate.

## 4. Results and Discussion

Rigid transform matrices (translation and rotation) for registration of the reference with the tested position have been estimated by the proposed ICP algorithms and with AlignRT. The investigated implementations specify the resulting coordinate transform for registration by different versions of matrices for homogenous coordinates. For direct comparison, these matrices have been transformed into a single representation for translation and rotation (see [12]).

Translational shift values of registration ${\hat{s}}_{d}$ in direction *d* yield directly from the offset part of the transform matrices.  Table 1 shows the results of registration together with the expected translation values *s*_*d*_. The translational registration error in direction *d* is then given by $e_{d}^{trans}=s_{d}-{\hat{s}}_{d}$.

The total registered rotation is composed by a series of three rotations *r* = {roll, pitch, yaw} around *x*-, *y*-, and *z*-axes, respectively, in the directions according to Figure 1(b), each quantified by the Euler angles ${\hat{\alpha }}_{r}=\{{\hat{\alpha }}_{roll},{\hat{\alpha }}_{pitch},{\hat{\alpha }}_{yaw}\}$. The rotatory registration error is $e_{r}^{rot}={\hat{\alpha }}_{r}-\alpha_{r}={\hat{\alpha }}_{r}$ for *α*_*r*_ = 0 because the measurement phantoms have not been rotated.

We assumed a maximally allowed absolute registration error of *e*_*d*_^trans^_max_ = 1 mm for translation and of *e*_*d*_^rot^_max_ = 0.5° for rotation which are quite tough values in radiotherapy and marked entries with |*e*_*d*_^trans^| > *e*_*d*_^trans^_max_ or |*e*_*d*_^trans^| > *e*_*d*_^rot^_max_ boldface. Other works set the allowable tolerance a bit higher (e.g., [8] to 1 mm/1° and [11] to 3 mm/3°), but working with rigid phantoms without motion motivates our stricter limits.

**Figure 4:**
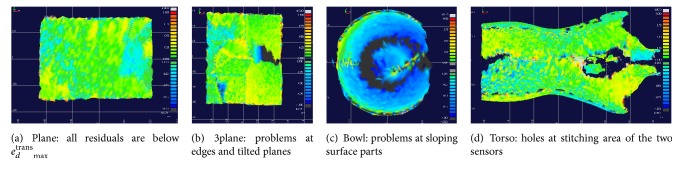
Residuals of surface pairs shifted by *s*_*d*_ = 10 mm in direction *d* = *z* of the studied phantoms captured by the optical sensor AlginRT and aligned by the Wilm approach.

Figure 4 shows as an example one of the best results of aligned surfaces with the reference surface for a shift of *s*_*d*_ = 10 mm in direction *d* = *z* using the Wilm approach. The residuals have been estimated by triangulation of the surfaces and color-coded displaying the distances in *z* direction. It becomes clear that although the translational and rotational parameters are within the limits this does not hold for all points of the surfaces. There are problems especially at sloping surface parts, at edges, and at the stitching area of left and right optical sensors which explains the remaining deviations after applying the ICP algorithm.

Table 2 summarizes Table 1 with regard to adhering the limits *e*_*d*_^trans^_max_ and *e*_*d*_^rot^_max_. As expected with the plane phantom placed in parallel to the *xy* plane a safe registration is only possible in *z* direction and fails in *x* and *y* direction for all ICP algorithms. For the other three phantoms 3plane, bowl, and torso, only the Wilm algorithm registers safely for the translational parameters. No algorithm has problems with the rotatory parameters for any phantom except Wilm which interestingly fails for the torso phantom for pitch and yaw and AlignRT for yaw of the bowl phantom.

Table 3 compares some important properties and results of the four tested algorithms that have been applied to four different test objects (phantoms) differently shifted relative to an original position. The algorithms use different methods to compute the rigid transformation matrix (translation and rotation) between two point clouds, as described in Section 2.3 as the result of registration.

Main operational principles of the algorithms are summarized; their processing speed and accuracy give information on their suitability for registration of our selected phantoms. Main differences consist in the method for the closest point search, the weighting, the error metric, and the method for minimization. Only Wilm uses kd-tree search which is much more efficient than full search. Only Bergström applies distance-based weighting. None of the open source algorithms includes rejection. Among the open source algorithms, only Wilm uses point-to-plane metric whereas all other apply a point-to-point criterion. The AlignRT registration results look similar to the Wilm implementation. This supports the assumption that similar principles are used by this proprietary program.

The average processing time for each algorithm is also qualitatively given. It varies between fastest processing (which was about a few seconds) and slowest processing (which was about 3 minutes) for the registration by the ICP algorithm on a standard computer (Intel Core i7, 64-bit Windows) in Matlab. A more detailed evaluation of processing speed is not given because we do not expect that the chosen algorithms are implemented in an optimal way. This may be different for the commercial implementation of AlignRT. Renoald performed best with regard to processing speed. Wilm and AlignRT show acceptable speed in the same range. Kroon is slow and Bergström is very slow in the investigated implementation and would not be acceptable in clinical routine.

For offline verification, speed plays a less important role as long as the registration takes only seconds of time. Therefore those implementations indicated by + or ++ can be considered acceptable in the intended application (see Section 3.2). In tracking applications, when even the registration is done online, the speed of the algorithms matters much more and the patient alignment can be verified and corrected on the fly by moving the treatment table or adapting the irradiation. But in this case, the algorithm needs much less iterations because the position differences from time step to time step are much smaller compared to the first alignment in the static case.

In Table 3, an overall assessment of the expected registration error between expected shift and the translation calculated by the ICP algorithms is given. Translation in *x* and *y* direction was omitted for the plane phantom because no registration was possible due to the missing structure in viewing field and therefore only the translation in the *z* direction is specified.

One observation from the experiments is that the distance of shift does not affect the registration accuracy much. Also the required time for convergence is not really affected, obviously because the algorithms adapt their step size according to the gradient.

Much more important are the structure and curvature of the surfaces to be aligned. With an ambiguous surface the error surface has flat areas where ICP algorithms are likely to stick in a local minimum. Registration fails in this case to align the surfaces [25].

Wilm's implementation shows the best results among the studied ICP algorithms for translational registration. The reason for that is obviously the use of the point-to-plane error metric which is the main difference to the other algorithms all failing with the above specified accuracy demands. Interestingly, Wilm fails with rotatory registration for the torso phantom. Possibly the normal parameter of the point-to-plane error metric has disadvantages in this case. Similar happens for the AlignRT implementation, but for the bowl phantom.

## 5. Conclusion

In the paper, different unconstrained ICP algorithms have been compared for real (noisy) data produced by an optical sensor as part of a Tomotherapy HD system. Registration has to deal with mainly two difficulties: the deficiencies of the sensor (noise) and the ambiguities resulting from the shape of the measured object. Reference [3] found accuracies better than 1 mm and 0.5° for the used mannequin torso phantom with the proprietary registration software of the AlignRT system. We could show that such accuracies are only possible for well curved surfaces whereas gross errors may occur for registration of other not uniquely shaped surfaces and are not much effected by the chosen ICP registration algorithm.

The results show that obviously standard ICP algorithms only considering point cloud or surface data are too unreliable to serve as single verification tool of correct patient settlement. Of course, large correction values calculated by ICP registration give a clear hint that positioning is incorrect whereas the opposite case does not hold: as small value is no guarantee for correct alignment. Depending on the curvature of the actually captured surface parts, small ICP registration correction values are estimated even with wrong positioning because the ICP algorithm sticks in local minima. The registration information in parallel to the main orientation of the surface is only helpful in the case of unique surface structure. A safe registration useful for setup correction mostly yields perpendicular to the main orientation of the surface. Therefore, the result of ICP registration can only support the expertise of the clinical personnel as an additional tool for the positioning of the patient with regard to the treatment machine.

To improve the probability of reaching a correct deviation minimum without fiducial markers other variants of ICP algorithm including additional criteria such as colors, normals, and curvatures [25] may be applied. The hardware of the optical sensor supports this because an additional camera for capturing texture data is included in each measurement unit. But according to [3], although calibrated together with the stereo cameras, it can be only used for virtually projecting texture data on the captured surfaces, but not to support registration. Particularly, uncertainties of the registration in *x*-*y* direction could be reduced by this information.

Ongoing work is done on the estimation of confidence values of registration. Depending on curvatures characteristics of the treated regions an estimation of the reliability of a registration could be given. Also alternative registration approaches to surface registration, such as probabilistic methods [18], seem promising to improve the results and worthy of further investigation.

## Figures and Tables

**Table 1:** Registered translations ${\hat{s}}_{x}$, ${\hat{s}}_{y}$, and ${\hat{s}}_{z}$ and rotations ${\hat{\alpha }}_{roll}$, ${\hat{\alpha }}_{pitch}$, and ${\hat{\alpha }}_{yaw}$ from the investigated sample implementations of the ICP algorithm for the tested phantoms plane, 3plane, bowl, and torso and treatment table shifts *s*_*d*_ in different directions *d* = {*x*, *y*, *z*}. Translations with an absolute translational registration error |*e*_*d*_^trans^| > *e*_*d*_^trans^_max_ = 1 mm and rotations with an absolute registration error *e*_*r*_^rot^ > *e*_*d*_^rot^_max_ = 0.5° are marked boldface.

Phantom	Shift		Registration					
			Translation/mm			Rotation/°		
	*s*_*d*_/mm	*d*	${\hat{s}}_{x}$	${\hat{s}}_{y}$	${\hat{s}}_{z}$	${\hat{\alpha }}_{roll}$	${\hat{\alpha }}_{pitch}$	${\hat{\alpha }}_{yaw}$
*Wilm algorithm*								

Plane	0.5	*z*	0.4282	0.0087	0.5482	−0.0001	−0.0012	0.0000
	1.0	*z*	**1.4503**	0.4656	1.0627	0.0000	−0.0123	0.0000
	10.0	*z*	0.4760	0.8346	10.0927	0.0002	−0.0022	−0.0005
	20.0	*z*	0.0827	**1.1255**	20.0975	−0.0001	0.0213	−0.0007

3plane	0.5	*z*	−0.0100	0.1440	0.5325	−0.0000	0.0001	−0.0003
	1.0	*z*	−0.0395	0.1362	1.0882	0.0001	0.0004	0.0001
	10.0	*z*	−0.1475	0.1217	10.0550	0.0003	0.0001	−0.0003
	20.0	*z*	−0.0665	−0.0903	20.9796	0.0005	0.0012	−0.0006
	10.0	*y*	−0.1237	9.9743	0.0157	0.0001	0.0004	0.0001
	−10.0	*y*	−0.1991	−9.6955	0.1602	0.0002	−0.0001	0.0004
	10.0	*x*	9.8886	0.3100	0.1109	0.0013	−0.0004	0.0001

Bowl	0.5	*z*	0.0749	0.0702	0.4491	−0.0006	0.0013	−0.0000
	1.0	*z*	0.0596	0.1477	0.9348	−0.0010	−0.0018	−0.0011
	10.0	*z*	−0.0084	0.0054	10.1927	0.0061	0.0121	0.0004
	20.0	*z*	0.3618	0.0843	20.0056	0.0005	0.0382	−0.0001
	10.0	*y*	−0.1668	9.9264	0.1325	0.0035	−0.0678	−0.0033
	−10.0	*y*	0.0885	−9.7892	−0.3034	0.0004	0.0246	−0.0006
	10.0	*x*	9.9217	−0.0522	0.0799	−0.0014	−0.0400	−0.0024
	−10.0	*x*	−9.8553	0.1800	−0.0533	−0.0005	0.0854	−0.0005

Torso	0.5	*z*	−0.0239	−0.0895	0.5171	0.0033	**1.5783**	**−1.5785**
	1.0	*z*	−0.0986	−0.1490	1.0508	0.0022	**1.6005**	**−1.6007**
	10.0	*z*	0.0303	0.1723	9.9136	0.0090	**1.1490**	**−1.1480**
	20.0	*z*	0.0995	0.1012	19.8599	0.0017	**0.8377**	**−0.8362**
	10.0	*y*	0.1819	9.9194	−0.1830	0.0009	**0.9571**	**0.9572**
	−10.0	*y*	−0.1553	−9.5344	0.0316	0.0020	**−1.4725**	**1.4724**
	10.0	*x*	10.1230	−0.0174	−0.0374	0.0047	**1.5300**	**−1.5299**
	−10.0	*x*	−9.8724	−0.0488	−0.0401	0.0034	**−1.6261**	**1.6263**

*Kroon algorithm*								

Plane	0.5	*z*	−0.4031	−0.1523	0.5499	−0.0001	−0.0001	−0.000
	1.0	*z*	−0.9254	−0.2737	1.0675	0.0002	0.0005	−0.0001
	10.0	*z*	1.3403	**1.2951**	10.0872	0.0002	−0.0015	−0.0004
	20.0	*z*	**−3.7160**	0.7362	20.0891	−0.0001	0.0007	−0.0008

3plane	0.5	*z*	0.0023	−0.1280	0.3746	−0.0005	0.0000	0.0000
	1.0	*z*	−0.0470	−0.2840	0.8252	−0.0001	0.0003	0.0001
	10.0	*z*	−1.5692	−5.8559	**7.3710**	−0.0125	−0.0045	−0.0043
	20.0	*z*	**1.0803**	**−4.9535**	**18.1806**	1.0803	0.0014	−0.0021
	10.0	*y*	0.0793	**6.5978**	**−1.3214**	−0.0066	−0.0004	−0.0003
	−10.0	*y*	−0.1597	**−6.6084**	**−1.3826**	0.0062	0.0004	0.0006
	10.0	*x*	**0.7600**	−0.0439	0.0272	0.0002	0.0008	−0.0018

Bowl	0.5	*z*	0.0818	−0.0440	0.2600	0.0009	0.0004	−0.0004
	1.0	*z*	0.0572	−0.0864	0.5162	0.0015	0.0002	−0.0004
	10.0	*z*	**−1.4703**	−0.2302	**8.9576**	0.0087	0.0022	−0.0420
	20.0	*z*	**−1.2950**	−0.1217	19.0383	0.00206	0.0035	−0.0554
	10.0	*y*	−0.2905	**8.8071**	0.0682	0.0369	−0.0581	−0.0027
	−10.0	*y*	0.2614	**−8.6043**	0.3671	−0.0368	0.0533	0.0006
	10.0	*x*	9.4381	−0.4111	0.0864	0.0023	0.0202	−0.0048
	−10.0	*x*	−9.1155	0.5139	0.1217	0.0004	−0.0187	0.0473

Torso	0.5	*z*	0.3474	**1.4598**	0.1776	−0.0003	0.0003	−0.0128
	1.0	*z*	0.3474	**1.5383**	0.0214	−0.0012	0.0013	−0.0214
	10.0	*z*	0.0014	0.2854	10.4856	0.0004	0.0018	−0.0006
	20.0	*z*	0.2456	0.8967	20.8858	0.0016	0.0010	−0.0012
	10.0	*y*	0.1391	**0.8154**	0.5305	0.0032	−0.0002	−0.0007
	−10.0	*y*	0.0276	−0.7041	−0.3240	−0.0025	0.0002	−0.0003
	10.0	*x*	10.7852	0.6038	0.0172	0.0002	0.0026	−0.0353
	−10.0	*x*	−10.6538	**−1.5451**	0.2848	0.0005	−0.0080	0.0327

*Renoald algorithm*								

Plane	0.5	*z*	−0.3951	−0.1486	0.5499	−0.0001	−0.0001	−0.0000
	1.0	*z*	−0.8802	−0.2661	1.0675	0.0000	0.0006	−0.0001
	10.0	*z*	**1.2549**	**1.1879**	10.0882	0.0002	−0.0010	−0.0005
	20.0	*z*	**−2.0547**	−0.4494	20.0903	−0.0001	−0.0040	−0.0006

3plane	0.5	*z*	0.0041	−0.1311	0.3722	−0.0005	0.0000	0.0000
	1.0	*z*	−0.0469	−0.2864	0.8242	−0.0006	0.0003	0.0001
	10.0	*z*	−0.0744	**−4.5305**	**8.2095**	−0.0099	−0.0003	−0.0039
	20.0	*z*	0.9315	**−6.7487**	**16.8058**	−0.0134	0.0058	0.0033
	10.0	*y*	0.1794	**4.0410**	**−1.1857**	−0.0124	−0.0004	−0.0002
	−10.0	*y*	−0.2608	**−5.4906**	**1.3925**	0.0088	0.0009	0.0005
	10.0	*x*	**0.7315**	−0.0448	0.0321	0.0002	0.0009	−0.0018

Bowl	0.5	*z*	0.0818	−0.0440	0.2600	0.0009	0.0004	−0.0004
	1.0	*z*	0.0571	−0.0831	0.5143	0.0015	0.0003	−0.0004
	10.0	*z*	**−1.2139**	−0.1348	**8.8075**	0.0069	0.0014	−0.0393
	20.0	*z*	**−1.5751**	−0.1130	**18.7352**	0.0056	0.0045	−0.0556
	10.0	*y*	−0.4060	**7.5070**	−0.0636	0.0356	−0.0402	−0.0014
	−10.0	*y*	0.3518	**−8.5498**	0.2583	−0.0358	0.0521	0.0009
	10.0	*x*	**8.8252**	−0.2963	−0.1059	−0.0000	0.0147	−0.0470
	−10.0	*x*	**−8.9940**	0.4230	0.1382	0.0007	−0.0171	0.0477

Torso	0.5	*z*	0.2920	0.4220	0.7578	0.0005	0.0012	−0.0321
	1.0	*z*	0.2378	0.4817	1.0779	0.0003	0.0009	−0.0315
	10.0	*z*	−0.0037	0.2426	10.4506	0.0001	0.0018	−0.0006
	20.0	*z*	0.3008	−0.1655	**18.7455**	−0.0011	0.0022	−0.0001
	10.0	*y*	0.1177	**0.7804**	0.4367	0.0028	−0.0002	−0.0004
	−10.0	*y*	0.0045	−0.6522	−0.2843	−0.0022	0.0001	−0.0001
	10.0	*x*	10.4139	0.4050	0.0860	0.0004	0.0015	−0.0327
	−10.0	*x*	−10.4768	−0.6117	0.1427	−0.0003	−0.0019	0.0334

*Bergström algorithm*								

Plane	0.5	*z*	0.0818	−0.0440	0.2600	0.0009	0.0004	−0.0003
	1.0	*z*	−0.9302	−0.2768	1.0675	0.0000	0.0005	−0.0001
	10.0	*z*	**−4.8742**	−0.3023	10.0866	0.0002	0.0013	−0.0008
	20.0	*z*	**−3.7203**	0.7381	20.0894	−0.0000	0.0001	−0.0008

3plane	0.5	*z*	0.0023	−0.12880	0.3746	−0.0005	0.0003	0.0000
	1.0	*z*	−0.0470	−0.2840	−0.8252	−0.0060	0.0003	0.0001
	10.0	*z*	**−8.9059**	**4.1357**	**11.6053**	0.0083	−0.0080	−0.0024
	20.0	*z*	**1.0816**	**−4.9472**	**18.1816**	−0.0095	0.0014	−0.0021
	10.0	*y*	**−2.1412**	**13.7773**	**1.6485**	0.0079	0.0060	0.0009
	−10.0	*y*	−0.1596	**−6.6095**	**1.3820**	0.0062	0.0004	0.0006
	10.0	*x*	**8.4082**	**2.8181**	**1.2289**	0.0064	−0.0053	−0.0034

Bowl	0.5	*z*	0.0818	−0.0440	0.2600	0.0009	0.0004	−0.0004
	1.0	*z*	0.0527	−0.0803	0.5211	0.0016	0.0003	−0.0004
	10.0	*z*	−0.1868	−0.0644	10.2913	−0.0002	−0.1752	0.0064
	20.0	*z*	0.6199	−0.0374	20.2321	0.0092	−0.1716	0.0172
	10.0	*y*	0.0319	10.0246	−0.4303	0.0058	−0.1689	0.0073
	−10.0	*y*	0.4347	−9.4345	0.9152	−0.0312	−0.1553	0.0238
	10.0	*x*	9.9406	**−2.1170**	0.6590	0.0190	0.0004	0.0001
	−10.0	*x*	−10.3737	**−1.6094**	0.3040	0.0133	0.0020	−0.0003

Torso	0.5	*z*	0.3383	**−2.5946**	0.8076	0.0004	0.0001	−0.0128
	1.0	*z*	0.1906	**−2.6231**	0.9682	−0.0000	0.0000	−0.0121
	10.0	*z*	0.0014	0.2854	10.4856	0.0040	0.0018	−0.0006
	20.0	*z*	−0.4539	−0.3521	19.4238	0.0008	0.0023	0.0150
	10.0	*y*	0.5342	**8.9866**	0.3959	0.0007	−0.0020	−0.0169
	−10.0	*y*	−0.1569	−8.6553	1.2477	−0.0070	0.0020	0.0012
	10.0	*x*	10.4504	**−2.5372**	0.4949	0.0003	0.0003	−0.0131
	−10.0	*x*	−10.2027	**2.5363**	0.3930	−0.0003	−0.0002	0.0120

*AlignRT algorithm*								

Plane	0.5	*z*	0.1	0.3	0.0	0.0	0.0	0.0
	1.0	*z*	0.2	0.1	0.5	0.0	0.0	0.1
	10.0	*z*	0.3	0.0	10.1	0.0	0.0	0.1
	20.0	*z*	0.2	**1**.**2**	20.1	0.0	0.0	0.2

3plane	0.5	*z*	0.0	0.1	0.5	0.0	0.0	0.0
	1.0	*z*	0.1	0.1	1.1	0.0	0.0	0.0
	10.0	*z*	0.0	0.2	10.2	0.0	0.0	0.0
	20.0	*z*	0.0	0.1	20.1	0.0	0.0	0.1
	10.0	*y*	0.2	10.2	0.2	0.0	0.0	0.0
	−10.0	*y*	0.1	−9.8	0.1	0.0	0.0	0.0
	10.0	*x*	9.8	0.2	0.1	0.0	0.0	0.0

Bowl	0.5	*z*	0.1	0.1	0.5	0.0	0.0	0.0
	1.0	*z*	0.1	0.2	1.1	0.0	0.0	0.0
	10.0	*z*	0.1	0.2	10.0	0.1	0.0	**1**.**2**
	20.0	*z*	0.3	0.3	19.9	0.1	0.0	**1**.**6**
	10.0	*y*	0.2	10.3	0.2	0.0	0.1	**1**.**6**
	−10.0	*y*	0.7	−9.8	0.2	0.1	0.1	**2**.**7**
	10.0	*x*	10.1	0.2	0.2	0.0	0.1	0.1
	−10.0	*x*	−10.0	0.5	0.2	0.0	0.0	**1**.**7**

Torso	0.5	*z*	0.1	0.0	0.6	0.0	0.0	0.0
	1.0	*z*	0.1	0.0	1.1	0.0	0.0	0.0
	10.0	*z*	0.1	0.0	9.9	0.1	0.0	0.1
	20.0	*z*	0.2	0.1	19.8	0.0	0.1	0.1
	10.0	*y*	0.2	10.1	0.2	0.0	0.0	0.0
	−10.0	*y*	0.2	−9.8	0.1	0.1	0.0	0.0
	10.0	*x*	10.0	0.2	0.1	0.0	0.0	0.0
	−10.0	*x*	−9.9	0.1	0.0	0.0	0.0	0.0

**Table 2:** Summary of registration success and fail for translations (*x*, *y*, *z*) and rotation (rot); + denotes success when the specified misalignment threshold is deceeded and, otherwise, labels the failing when the threshold is exceeded.

		Algorithm																			
		Wilm				Kroon				Renoald				Bergström				AlignRT			
Motion		*x*	*y*	*z*	rot	*x*	*y*	*z*	rot	*x*	*y*	*z*	rot	*x*	*y*	*z*	rot	*x*	*y*	*z*	rot
Phantom	Plane	−	−	+	+	−	−	+	+	−	−	+	+	−	−	+	+	+	−	+	+
	3plane	+	+	+	+	−	−	−	+	−	−	−	+	−	−	−	+	+	+	+	+
	Bowl	+	+	+	+	−	−	−	+	−	−	−	+	+	−	+	+	+	+	+	−
	Torso	+	+	+	−	+	−	+	+	+	−	+	+	+	−	+	+	+	+	+	+

+: |*e*_*d*_^trans^| < *e*_*d*_^trans^_max_ and |*e*_*r*_^rot^| < *e*_*d*_^rot^_max_; −: |*e*_*d*_^trans^| > *e*_*d*_^trans^_max_ and |*e*_*r*_^rot^| > *e*_*d*_^rot^_max_.

**Table 3:** Overall assessment of the tested ICP algorithms.

Property	Algorithm				
	Wilm	Kroon	Renoald	Bergström	AlignRT
Closest point search	kd-tree	Full	Full	Full	—^*a*^

Weighting	None	None	None	Distance-based	—^*a*^

Rejection	None	None	None	None	—^*a*^

Error metric	Point-to-plane	Point-to-point	Point-to-point	Point-to-point	Point-to-plane

Minimization	Linearization of rotation matrix	Global search	SVD	Levenberg-Marquardt	—^*a*^

Speed^b^	+	−	++	− −	+

Max. |*e*_*r*_^rot^|	<1.0 mm	>1.0 mm	>1.0 mm	>1.0 mm	<1.0 mm
Max. |*e*_*d*_^trans^|	>0.5°	<0.5°	<0.5°	<0.5°	>0.5°

^a^Unknown.

^b^++: very fast; +: fast; −: slow; − −: very slow.
